# College and Hospital Notes

**Published:** 1907-04

**Authors:** 


### COLLEGE AND HOSPITAL NOTES.

The Riverside Accident Hospital Dispensary, Buffalo, was the recipient of a benefit given at Orient Hall early in March, 1907, which was attended by about 200 persons. The guests were received by Dr. Lillian Craig Randall and Mrs. Charles P. Chapin.

The Buffalo Children's Hospital was recently the recipient of $10,000, contributed to the endowment fund by Mr. William A. Rogers. It has been previously announced in these columns that Miss Martha T. Williams last summer gave the present site of the hospital and the building now used for patients. Mrs. Charles W. Pardee gave the funds for a new building some weeks ago,, which will cost $75,000 to $100,000. The new structure will be a fireproof building, work upon which will be started very soon.

